# Prevalence of impaired glucose tolerance and other types of dysglycaemia among young twins and singletons in Guinea-Bissau

**DOI:** 10.1186/s12902-016-0126-6

**Published:** 2016-08-04

**Authors:** Ditte Egegaard Hennild, Morten Bjerregaard-Andersen, Luis Carlos Joaquím, Kaare Christensen, Morten Sodemann, Henning Beck-Nielsen, Dorte Møller Jensen

**Affiliations:** 1 Bandim Health Project, INDEPTH Network, Apartado 861, 1004 Bissau Codex, Guinea-Bissau; 2 Department of Infectious Diseases, Odense University Hospital, Sdr. Boulevard 29, 5000 Odense C, Denmark; 3 Department of Endocrinology, Odense University Hospital, Kløvervænget 6, 5000 Odense C, Denmark; 4 Research Center for Vitamins and Vaccines (CVIVA), Statens Serum Institute, Artillerivej 5, 2300 Copenhagen S, Denmark; 5 Guinean Diabetes Association (ANDD), Bissau, Guinea-Bissau; 6 The Danish Twin Registry, Epidemiology, Institute of Public Health, University of Southern Denmark, J.B. Winsløwsvej 9, 5000 Odense C, Denmark; 7 Department of Clinical Genetics, Odense University Hospital, Sdr. Boulevard 29, 5000 Odense C, Denmark; 8 Department of Clinical Biochemistry and Pharmacology, Odense University Hospital, Sdr. Boulevard 29, 5000 Odense C, Denmark; 9 Elite Research Centre for Medical Endocrinology, Odense University Hospital, Sdr. Boulevard 29, 5000 Odense C, Denmark; 10 The Research Unit of Gynaecology and Obstetrics, Odense University Hospital, Sdr. Boulevard 29, 5000 Odense C, Denmark

**Keywords:** Twins, Fetal origins hypothesis, Impaired glucose tolerance, Impaired fasting glucose, Diabetes, Sub-Saharan Africa, Low birth weight

## Abstract

**Background:**

Twins may be at increased risk of dysglycaemic disorders due to adverse fetal conditions. Data from Africa regarding this association is limited. We studied impaired glucose tolerance (IGT) and other types of dysglycemia among twins and singletons in Guinea-Bissau.

**Methods:**

The study was conducted from February 2011 until March 2012 at the Bandim Health Project, a health and demographic surveillance system site in the capital Bissau. Twins (*n* = 209) and singletons (*n* = 182) were recruited from a previously established cohort. Oral glucose tolerance tests (OGTT) were performed, along with anthropometrics and collection of clinical and dietary data.

**Results:**

Median age was 16.6 and 14.2 years between twins and singletons, respectively (*P* = 0.08). Mean birth weight was 2410 vs. 3090 g, respectively (*P* < 0.001). Twins had higher median fasting- and two hour capillary plasma glucose, 5.4(3.2–8.2) vs. 5.0(3.2–11.5) mmol/L (*P* < 0.001) and 6.8(3.4–11.3) vs. 6.2(3.2–12.1) mmol/L (*P* < 0.001), respectively, compared to singletons. The prevalence of IGT was 2.5 % (5/209) vs. 3.5 % (6/182) (RR = 0.73, 95 % CI: 0.20–2.64). 12 % (25/209) of twins had impaired fasting glucose (IFG), compared to 3.5 % (6/182) of singletons (3.63, 1.53–8.62). Dysglycemia (IGT and/or IFG or overt diabetes) was found in 17 % (35/209) vs. 9 % (16/182) (1.90, 1.08–3.37), respectively.

**Conclusions:**

Twins had higher glucose levels in both the fasting and postprandial state. This may indicate a detrimental effect of the twin fetal environment on glucose metabolism later in life, a result contrary to Scandinavian register studies. The IGT burden was low in this young age group and the risk was similar in twins and singletons.

**Electronic supplementary material:**

The online version of this article (doi:10.1186/s12902-016-0126-6) contains supplementary material, which is available to authorized users.

## Background

According to the fetal origins hypothesis an adverse fetal environment predisposes to cardiovascular and dysmetabolic disorders later in life [1–3]. Birth weight is often used as a marker of the intrauterine environment, and studies show that low birth weight (LBW) is associated with type 2 diabetes mellitus (DM) [4–6]. As twins are frequently born with LBW, they may be at higher risk of developing metabolic diseases related to glucose homeostasis, compared to singletons [7]. Such an association would be of great interest in Sub-Saharan Africa, where twinning is common [8] and the DM prevalence is increasing rapidly [9, 10].

Twinning and metabolic disorders have been investigated in high income settings. Clinical studies from Denmark showed that twins had a higher burden of insulin resistance and abdominal obesity [7], while an Italian study observed a higher rate of metabolic syndrome [11]. Another Danish population based study showed an increased risk of type 2 DM in elderly Danish twins [12]. A large scale Danish register study did, however, not find a higher DM burden among twins [13], a finding replicated later in a Swedish register study [14].

Yet, the above results may not be applicable to Sub-Saharan Africa, where the fetal and post-natal environment experienced by twins and singletons could be different. Here, large populations are currently undergoing considerable transition in life style, i.e., to a more westernized calorie dense nutrition [4]. Combined with a high incidence of LBW among African children due to fetal undernutrition [15], the dysmetabolic consequences of fetal programming could be more common in Sub-Saharan Africa, as the mismatch between fetal and adult nutritional environments seem to be particularly harmful [4]. Furthermore, malaria and HIV could have a detrimental effect on the intrauterine environment [4, 16], and changes could also arise due to genetic background [17] and seasonal variation food intake [18].

Impaired glucose tolerance (IGT) is a pre-diabetic condition, with many individuals developing DM over 5–10 years [19, 20]. In Africa, the prevalence of IGT has been estimated to between 2 and 13 % in various studies [10, 21–24], with the burden rapidly increasing in several parts [9]. The main objective of the present investigation was describing the prevalence of IGT in a well-defined cohort of young twins and singletons in Guinea-Bissau, and to determine whether twins were at a higher risk of IGT [8].

## Methods

### Study population

The study was carried out at the Bandim Health Project (BHP), a health and demographic surveillance system (HDSS) site in Bissau, the capital of Guinea-Bissau. The BHP is a member of the “International Network for the Demographic Evaluation of Populations and Their Health in Developing Countries” (INDEPTH) and follows a population of 100,000 individuals. All are registered with an ID-number, age, sex, ethnic group and socio-economic characteristics. Information is available regarding early childhood morbidity, nutrition and vaccination status. Twin status is recorded as well, and the HDSS database is updated through regular censuses. A diabetes notification system has been implemented.

Guinea-Bissau is ranking among the poorest nations in the world [25]. It has experienced substantial political instability over the last decades, including civil war.

### Selection

Our study took place from February 2011 until March 2012, including during the rainy season from May to October. Participants were recruited from a large cohort, designed to investigate the prevalence of DM and metabolic syndrome among young twins and singletons [26]. Twins in the present sub-study were drawn by opportunity from the original cohort, i.e., a field assistant and a laboratory technician would re-visit the houses in the BHP study area in a random order, enrolling any of the previously participating twins available. For every twin enrolled, it was attempted to recruit a random singleton control from the previous study with roughly the same age. The number of singletons investigated was therefore lower, as sometimes a singleton control with similar age was not available for testing. Twins were enrolled during the entire study period, while singletons only from September 2011.

### Interview

All field interviews were conducted in the local language Portuguese Creole. Provided consent, a questionnaire was used to obtain information regarding family history of DM, alcohol consumption and smoking habits. Twin status was confirmed. Participants were asked if they suffered from a chronic disease and if so whether this was DM, hypertension, cardiovascular disease or other. We used a specific questionnaire concerning nutritional habits. It had not been validated previously, but was designed to cover the major types of food intake in Bissau.

### Physical examination

A manual sphygmomanometer was used for the blood pressure measurements (BP), with the person seated at rest and wearing light clothing. Weight was recorded using regularly calibrated standard bathroom scales, height by metallic measuring tape, with the person barefooted. Middle upper arm circumference (MUAC) was measured by non-stretchable measuring tape (TALC, Herts, United Kingdom). Waist circumference (WC) was measured at midpoint between the lower costal margin and the iliac crest, hip circumference (HC) at the widest point in the gluteal region.

A Harpenden Skinfold Caliper (Baty International, West Sussex, United Kingdom) was applied for triceps, biceps, subscapular and suprailiac skinfold measurements [27]. Body fat percentage (BF%) was computed for children and adults using previously established equations [28].

### Zygosity

Using data from the previous study, genetically established zygosity was available for a subset of twins. Zygosity had also previously been evaluated by physical similarity in twin pairs, with 89 % correctness [26].

### Oral glucose tolerance test (OGTT)

The OGTTs were performed after an overnight fast. The interview and physical examination were primarily carried out during the OGTTs. The majority of measurements were done by a HemoCue Glucose 201+ apparatus (HemoCue, Ängelholm, Sweden), which uses capillary blood and then automatically converts the results to plasma values. In 7 % of the twins and 21 % of the singletons an AccuChek Active apparatus (Roche Diagnostics, Indiana, USA) was used, due to shortage of HemoCue cuvettes. The AccuCheck Active also makes an automatic conversion to plasma values. The coefficients of variation (CV) for the two apparatuses are approximately 2 and 5 %, respectively.

For the OGTTs, a standard 75-g glucose solution was used in individuals with a weight above 43 kg, while for individuals below 43 kg a weight-adjusted glucose solution was used (1.75 g glucose/kg), in accordance with common procedures [29]. The standardized glucose containers were provided by Odense University Hospital, Denmark. Glucose measurements were done in fasting (zero hours), at one hour and at two hours.

### Definitions

The results were classified according to World Health Organization (WHO) 1998 criteria for glucose measurements on capillary plasma [29]. This was done since the most recent WHO report of 2006 did not include IGT criteria based on capillary plasma, only stating that the fasting values for venous and capillary plasma glucose are identical, whereas non-fasting capillary plasma yield higher glucose values as opposed to venous plasma. Hence, using capillary plasma values, IGT was defined as two hour glucose level of 8.9–12.1 mmol/L, DM as a fasting glucose ≥ 7.0 mmol/L or a two hour glucose of ≥12.2 mmol/L. A fasting glucose between 6.1 and 6.9 mmol/L was defined as impaired fasting glucose (IFG). Dysglycemia was defined as having any of IGT, IFG or DM, or a combination. Young individuals were defined as <16 years. Low birth weight (LBW) was birth weight below 2500 g.

### Statistics

Data were entered using dBase 5.0 software (dataBased Inc, Vestal, NY, USA). Statistical analyses were performed in STATA 11 (Stata Corporation, College Station, TX, USA). A *P* < 0.05 was considered significant.

Only participants with full OGTT results were analysed. The IGT burden was included as proportions, while median glucose levels for monozygotic (MZ) twins, dizygotic (DZ) twins and singletons were also calculated. Comparison of categorical outcomes between twins and singletons was done using Poission regression and expressed as relative risks (RR) and P-value. Linear regression was applied in case of comparison of continuous outcomes and expressed as differences (Diff) and P-value. In both models, the intra-pair relationship among twins was controlled for with the cluster function, using a unique *pair* number. For non-normally distributed continuous variables, the Wilcoxon rank-sum test was applied and values expressed as medians.

BMI was only calculated for individuals above 16 years. For those below, BMI z-scores were calculated, using current CDC growth references.

In a multivariate Poisson regression analysis, we adjusted for age, sex and high waist-to-hip ratio (above 0.90 for males and 0.85 for females) as potential confounders. Rainy season (May-October) was also added, in order to control for any related differences in food intake during this time of year.

As a secondary analysis, we furthermore restricted our metabolic analyses to twins where a singleton control with exact match (1:1) on date of birth was available. Age was here very similar for twins and singletons, with the only difference due to singletons on average being included slightly later.

## Results

### Enrolment

**Fig. 1:**
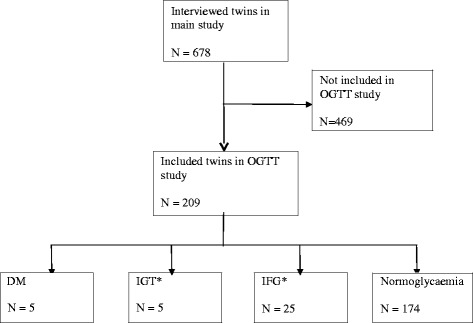
Inclusion of twins in the OGTT study. *One twin had co-existing IGT and IFG and has in this flowchart been classified as an IGT case

**Fig. 2:**
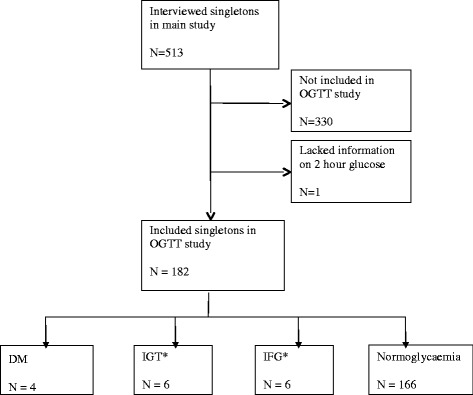
Inclusion of singletons in the OGTT study. *Two singletons had co-existing IGT and IFG and have in this flowchart been classified as IGT cases

The previous twin study interviewed 678 twins. Among these, an OGTT was performed in 209 confirmed twins (82 twin-pairs and 45 single twins), selected randomly by opportunity. In the previous study, 513 singleton controls had also been interviewed. Of these, an OGTT was performed in 183 confirmed singletons of similar age as the twins. However, one singleton lacked information on his two hour glucose and was excluded. The present study therefore included 182 singletons with OGTT results (Figs. 1 and 2).

### Baseline characteristics

Demographic and clinical data are displayed in Table 1. Twins tended to be slightly older than singletons, 16.6 years vs. 14.2 years (*P* = 0.08). The age range was 7–34 years for twins and 7-32 years for singletons. Among the twins, 116 (55.5 %) were adults aged 16 years or more. For the singletons, 82 (45.3 %) were adults. One singleton lacked information on age.

**Table 1:** Demographic and clinical characteristics for twins and singletons where OGTT results were available

	Twins *N* = 209	Singletons *N* = 182	RR or DIFF	*P*
Demographics				
Male sex; %	97/209 (46 %)	86/182 (47 %)	0.98 (0.79–1.22)	0.87
Age; years (T = 209, S = 181)	16.6 (7–34)	14.2 (7–32)		0.08
Children (<16 years); %	93/209 (44.5 %)	99/181 (54.7 %)	0.81 (0.64–1.03)	0.09
Adults (≥16 years): %	116/209 (55.5 %)	82/181 (45.3 %)	1.23 (0.98–1.54)	0.08
Ethnicity; %				
Balanta	23/209 (11.0 %)	14/182 (7.7 %)	1.42 (0.70–2.90)	0.33
Fula	14/209 (6.7 %)	21/182 (11.5 %)	0.58 (0.29–1.18)	0.13
Pepel	67/209 (32.1 %)	71/182 (39.0 %)	0.82 (0.60–1.13)	0.23
Mandinka	19/209 (9.1 %)	13/182 (7.1 %)	1.27 (0.59–2.75)	0.54
Other	86/209 (41.1 %)	62/182 (34.1 %)	1.21 (0.90–1.62)	0.21
Clinical data				
Chronic disease; %	12/209 (5.7 %)	8/178 (4.5 %)	1.28 (0.54–3.03)	0.58
Family history of DM^d^; %	15/183 (8.2 %)	8/176 (4.5 %)	1.80 (0.73–4.46)	0.16
Tobacco smoking; %^a^	5/116 (4.3 %)	4/80 (5.0 %)	0.86 (0.21–3.48)	0.84
Alcohol intake; %^a^	37/116 (31.9 %)	17/80 (21 %)	1.50 (0.89–2.53)	0.13
MUAC^e^; mm (T = 209, S = 181)	236 (38–354)	222 (140–342)		0.43
BMI/BMI z-score				
BMI; kg/m^2^ (T = 119, S = 85)^b^	20.6 (3.14)	21.7 (3.52)		0.02
BMI-z-score (T = 88, S = 96)^c^	−1.32 (1.41)	−1.09 (1.07)		0.23
WHR^f^ (T = 208, S = 180)	0.83 (0.42–1.00)	0.80 (0.35–1.38)		0.03
BF^g^; % (T = 207, S = 179)	12.1 (2.3–32.7)	11.1 (1.8–30.2)		0.02
Systolic BP^h^; mmHg (T = 201, S = 181)	104 (13)	107 (14)	3.5 (0.5–6.4)	0.02
Diastolic BP^i^; mmHg (T = 201, S = 181)	62 (11)	68 (12)	5.8 (3.3–8.3)	<0.001
Hypertension^b^	4/116 (3.5 %)	18/82 (22.0 %)	0.16 (0.05–0.45)	<0.001
Birth weight; g (T = 56, S = 46)	2410 (469)	3090 (397)	680 (500–860)	<0.001
Low birth weight (<2500 g); %	29/56 (52 %)	2/46 (4 %)	14.4 (3.6–57.3)	<0.001

Cells are n/N (%), mean (SD) or median (range). For continuous data, the total number of observations is listed as T for twins and S for singletons, as information was not complete for all variables

^a^Only adults were asked about smoking and alcohol habits

^b^Only for adults

^c^Only for young twins and singletons

^d^Diabetes Mellitus

^e^Middle Upperarm Circumference

^f^Waist-to-hip ratio

^g^Body Fat Percentage

^h^Systolic blood pressure

^i^Diastolic blood pressure

The male sex distribution was the same for twins and singletons, 46 % (97/209) vs. 47 % (86/182) (*P* = 0.87), respectively. Median BMI was lower for adult twins compared to singletons, 20.6 vs. 21.7 kg/m^2^ (*P* = 0.02), respectively. For young twins vs. singletons, no statistically significant difference was observed in BMI z-score (*P* = 0.23). Median WHR was higher for twins, 0.83 vs. 0.80 (*P* = 0.03), as was median BF%, 12.1 % vs. 11.1 % (*P* = 0.02).

Among 56 twins and 46 singletons for whom information on birth weight was available, 52 % of the twins were born with LBW, as opposed to 4 % among singletons (14.4, 3.6–57.3). The mean birth weight was 2410 g for twins and 3090 g for singletons (Diff = 680 g, 95 % CI: 500–860).

### Comparison with the previous study

When comparing the sampled OGTT twins with the previous twin study, the sex distribution was similar. The sampled OGTT twins were, however, slightly older, 16.6 vs. 15.3 years. For singletons, the sex distribution was also the same. The sampled OGTT singletons were, however, slightly younger compared to the previous study, 14.2 vs. 15.8 years.

### Metabolic outcomes

OGTT results are displayed in Table 2. Twins had higher median fasting glucose than singletons, 5.4 vs. 5.0 mmol/L (*P* < 0.001), respectively. After the OGTT, twins likewise had higher median glucose level, i.e., 6.8 vs. 6.2 mmol/L at two hours (*P* < 0.001). No difference was observed in glucose levels at one hour (*P* = 0.50).

**Table 2:** OGTT results for 209 twins and 182 singletons

	Twins	Singletons	RR (crude)^b^	*P* crude	RR (adjusted)^c^	*P* adj.
	*N* = 209	*N* = 182			*N* = 388	
Fasting glucose; mmol/L^a^	5.4 (3.2–8.4)	5.0 (3.2–11.5)		<0.001		
1-hour glucose; mmol/L^a^	7.1 (4.3–12.8)	7.0 (4.1–13.3)		0.50		
2-hour glucose; mmol/L^a^	6.8 (3.4–11.3)	6.2 (3.2–12.1)		<0.001		
IGT^d^	5/209 (2.5 %)	6/182 (3.5 %)	0.73 (0.20–2.64)	0.63	0.64 (0.18–2.32)	0.50
IFG^e^	25/209 (12 %)	6/182 (3.5 %)	3.63 (1.53–8.62)	0.004	4.47 (1.78–11.2)	<0.001
DM^f^	5/209 (2.4 %)	4/182 (2.2 %)	1.09 (0.30–3.98)	0.90	1.24 (0.35–4.42)	0.35
Dysglycaemia^g^	35/209 (17 %)	16/182 (9 %)	1.90 (1.08–3.37)	0.03	2.01 (1.12–3.61)	0.02

N is the total number of individuals in the adjusted analysis

^a^Glucose levels expressed as medians as non-normally distributed. Diff. could not be calculated here

^b^No adjustment

^c^Adjusted for age, sex, high WHR (>0.90 for males and 0.85 for females) and rainy season

^d^Impaired Glucose Tolerance

^e^Impaired Fasting Glucose

^f^Diabetes Mellitus

^g^The test person has any of IGT, IFG or DM, or a combination

In total, 2.5 % (5/209) of twins and 3.5 % (6/182) of singletons had IGT (RR = 0.73, 95%CI: 0.20–2.64) (*P* = 0.63). Twelve percent (25/209) of twins and 3.5 % (6/182) of singletons had IFG (3.63, 1.53–8.62) (*P* = 0.004). One twin and two singletons had concomitant IGT and IFG, but these were included as IGT (not IFG) cases in the statistical analysis. 2.4 % (5/209) of the twins and 2.2 % (4/182) of the singletons had DM, based the OGTT results alone (1.09, 0.30–3.98). In total, 17 % (35/209) of twins and 9 % (16/182) of singletons had some type of dysglycaemia (1.90, 1.08–3.37) (*P* = 0.03).

### Multivariate analysis

In a multivariate analysis, we adjusted the comparison of IGT, IFG, DM and dysglycaemia among twins vs. singletons for potential confounders such as age, sex, WHR and season. We found little effect on the risk of IGT, where the adjusted RR for twins was 0.64 (0.18–2.32). Regarding the risk of IFG among twins, the association became stronger after adjustment, i.e., the adjusted RR was 4.47 (1.78–11.2) (*P* < 0.001) (Table 2). For dysglycemia, the association remained fairly stable (2.01, 1.12–3.61).

### Effect of zygosity

**Fig. 3:**
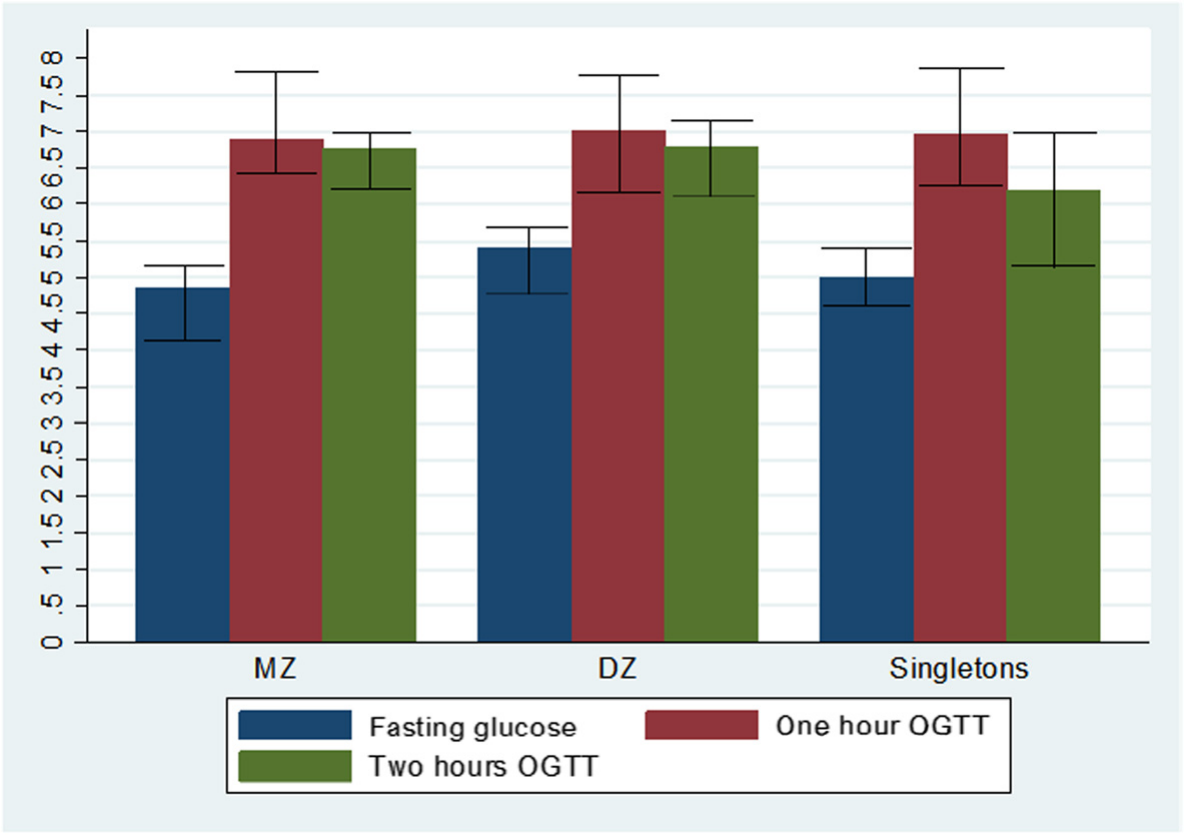
Median glucose levels (mmol/L) in fasting and at one and two hours during OGTT for the subset of twins where zygosity status was known. MZ twins (22/133), DZ twins (111/133), Singletons = 182/182. Interquartile ranges (25 and 75 % percentiles) are displayed. Note: This figure represents a subset of the data, i.e., only those with available zygosity and is therefore not directly comparable to the median values displayed in Table 2

From the previous study, genetically established zygosity was available for 133 twins, including 22 MZ and 111 DZ. At fasting, median glucose was significantly lower for MZ twins compared to DZ, 4.9 vs. 5.4 mmol/L (*P* = 0.003). No difference was observed during the OGTTs at one (*P* = 0.85) or two hours (*P* = 0.68) (Fig. 3).

### Nutrition

Nutritional intake based on the food questionnaire is displayed in the Supplemental Material (S1). Several variables differed for twins and singletons. Thus, twins were less likely to eat fish, meat and potatoes, compared to singletons (*P* < 0.05).

### Matched analysis

Eighty-seven twins had a singleton control with exactly the same date of birth. Among the 87 twins, the median age at enrolment was 15.2 years, compared to 15.4 years for the 87 singletons (*P* = 0.69). Median fasting glucose was 5.4 mmol/L for twins vs. 5.1 mmol/L for singletons (*P* = 0.009). At two hours OGTT, the median glucose levels were 6.7 vs. 6.3 mmol/L (*P* = 0.08) in the two groups, respectively. No difference was observed at one hour (*P* = 0.31).

### IGT, IFG and DM results comparing Hemocue vs. AccuCheck apparatuses

Fifteen twins and 38 controls were tested with an AccuChek Active apparatus. Out of these, one twin and one control were diagnosed with IGT. 194 twins and 144 singleton controls were tested with the HemoCue Glucose 201+ apparatus, out of which 4 twins and 5 controls had IGT. There was no difference in IGT prevalence between twins and controls tested with the HemoCue (*P* = 0.43) nor with the AccuCheck (*P* = 0.48).

Among the twins and singleton controls tested with the AccuCheck none had IFG or DM, whereas 25 twins vs. six controls had IFG (*P* < 0.001) and five twins vs. four controls had DM (*P* = 0.91) when tested with the HemoCue. In total, 34 twins and 15 controls had dysglycaemia when tested with the Hemocue (*P* = 0.06.), whereas only one twin and one control had dysglycaemia when tested with the AccuCheck (*P* = 0.48).

## Discussion

### Main findings

The IGT prevalence was low among young individuals in Guinea-Bissau, and it did not differ significantly between twins and singletons in both the crude and adjusted analysis. Twins did, however, have a significantly higher IFG burden, as well as a higher median glucose levels both in fasting and at two hours after glucose intake.

### Strengths and weaknesses

To our knowledge, this is the first study specifically performing OGTTs among twins in Sub-Saharan Africa. The fact that it was carried out within a well-established cohort likely improves data quality and ensures good follow-up possibilities.

When performing OGTTs, there is considerable *intra-subject* variability [19]. This affects the precision of our estimates, though the effect would likely be the same for twins and singletons. The majority of participants were investigated using a HemoCue device. Yet, for a small number an AccuCheck apparatus was used. This may have affected comparisons slightly. However, a sub-analysis examining twins and singletons tested with HemoCue and AccuCheck separately did not change the result significantly with regard to the prevalence of dysglycaemia.

For practical reasons, we conducted point of care testing using capillary blood glucose. This could be considered a limitation, since venous plasma glucose is the golden standard of glucose measurement [30]. According to a WHO report of 2006 capillary blood can, however, be used in under-resourced settings [30]. The CV of the two glucose measuring devices used of 2–5 % was considered acceptable and was likely less than the *intra-subject* variability.

The young age of the participants is also an important limitation as the risk of developing DM, IFG and IGT markedly increases with age. This was a consequence of the age of the overall HDSS. In the context of DM and age, it should be noted that many Type 1 DM patients die at an early age in Guinea-Bissau due to the cost and difficulties of insulin delivery.

Our main statistical analysis was not individually matched on age, but simply compared proportions of IGT in the two groups. However, in a sub-group analysis with exact 1:1 match on date of birth, a similar trend in glucose levels as in the overall sample was found.

Twins and singleton controls were not matched on sex either. Gender differences related to behavior and biology may affect glucose metabolism, particularly in adolescents [31, 32]. Yet, the sex distribution was very similar for twins and singletons, and inclusion of gender in the adjusted model did not change the estimates significantly.

Information regarding chronic diseases as well as family history of diabetes was based on interviews alone which likely makes the estimates more uncertain. Hence, these parameters were not included in the regression model. The increased attention on DM in Guinea-Bissau is fairly new, and during the field work our impression was that many participants at the time had little or no knowledge of the disease.

Birth weight was only available for 26 % of the participants due to many being born outside the BHP area. Birth weight was also often missing in the HDSS registrations from the 1980s and 1990s, and birth weight could therefore not be introduced in our regression model. Birth weight is a marker of the fetal environment, albeit only a crude one. Gestational age is a likely confounder, since twin pregnancies are known to have shorter gestation than singletons, often resulting in LBW [33]. Unfortunately, information regarding gestational age was unavailable. Previously, we have found that 41 % of twins in Guinea-Bissau were prematurely born (<37 weeks), compared to 23 % of singletons [34].

We did not have data regarding insulin sensitivity or beta cell function during the OGTTs. In the previous study, no difference was found in fasting insulin or HOMA insulin resistance between twins and singletons [26].

Finally, we did not have any data regarding maternal nutrition during pregnancy. This was a limitation, since factors such as maternal BMI and diet are likely of importance in fetal programming [31, 35].

### Consistency with previous findings

A number of investigations have assessed IGT in Africa, though with highly varying estimates, including between urban and rural areas. A study from neighboring Guinea-Conakry observed an IGT prevalence of 13 % in a middle-aged population [21]. A recent study from Kenya found a quite similar IGT burden of 12 %, though in a considerably younger population with mean age 38.6 years [22]. An older survey from Cameroon described an urban IGT prevalence of 2 % [23]. The overall IGT prevalence for the Africa region has been estimated to 7.3 % for adults [10]. In high income settings, IGT burdens of similar proportions have been observed [36, 37].

We did not observe a higher IGT burden among twins compared to singletons. This could suggest that LBW in twins – as a marker of adverse fetal conditions - is not strongly associated with IGT in our setting. In this context, it is important to consider whether different mechanisms lead to the LBW in twins vs. singletons. Twins could often be born with LBW due to preterm delivery and spatial limitations in utero, rather than due to an actual adverse fetal environment [13].

Our previous study found a prevalence of metabolic syndrome of 3.0 % for twins vs. 3.6 % of singletons in the same cohort, supporting that twins are not at higher risk of dysmetabolic disorders later in life. No difference was found in mean HbA1c either. Yet, as in the present investigation, higher fasting glucose and higher IFG prevalence were found in twins [26]. Adjusting for potential confounders such as age, sex, WHR and season caused the association between twin status and IFG to become even stronger.

Apart from the studies by our group, data on twinning and dysglycaemic disorders in Sub-Saharan Africa are not available, though investigations from high income settings have examined the association. Several smaller clinical studies have shown an association between twin status and dysglycemia [7, 12]. A Danish study found that MZ twins were particularly disadvantaged in terms of glucose metabolism [38], as they may experience even more adverse fetal conditions, e.g., due to placental vascular anastomoses [39]. However, the findings of the smaller clinical investigations have not been replicated in large scale Scandinavian register studies [13, 14], which found no differences in the DM burden between twins and singletons in adulthood. Furthermore, a study observed no intra-pair differences in glucose metabolism among extremely birth weight discordant MZ twins, indicating common genetic factors in the association between LBW and adult metabolic disease [40].

Importantly, we do not know how these findings relate to Guinea-Bissau, where twins have a very high perinatal mortality of 22 % [34]. Infant twin mortality is also twofold elevated, compared to singletons [41]. Birth weight below 2000 g thus remains an important risk factor for death during the first year of life [41]. A recent study demonstrated that twins were not hospitalized more often in Guinea-Bissau during infancy, despite elevated mortality [41]. A considerable “healthy survivor bias” is therefore likely, favoring twins in adulthood.

Also, adverse exposures during fetal life could be markedly different in Africa. Thus, in our setting around 13 % of all children are born with LBW [26], with twins accounting for 20–23 % of those [42]. This indicates that sub-optimal nutrition during pregnancy is common in Guinea-Bissau. Of particular concern in Africa is currently the combination of a resource poor fetal environment, followed by a nutrient rich diet later in life due to changes in type of food intake and availability in the populations, as this may predispose to DM and other metabolic disorders [4]. Data regarding nutritional transition in relation to DM are not available from Guinea-Bissau, but the tendency would likely be similar. Moreover, malaria - which is endemic across Africa - has been associated with both reduced fetal growth and elevated post-natal blood pressure [16], as has maternal HIV infection [43], access to antenatal care and socio-economic factors [44]. Such exposures could influence our glucose results, especially if correlated to twin status. Interestingly, singletons had higher blood pressure than twins.

In the subset of twins where zygosity status was known, MZ twins did not have higher glucose levels than DZ twins. This was somewhat surprising, as MZ twins in theory should experience an even more adverse fetal enviroment. A study from the main maternity ward in Bissau found a MZ twinning rate of 3.4/1000 [34].

We collected basic information regarding the nutritional intake. We found that singletons had a more frequent intake of fish, potatoes and meat. Twins had a more frequent intake of deep fried and oily food on a weekly basis, whereas singletons had a higher intake on a daily basis. There was no difference in the intake of vegetables, sweets and sweet beverages. Thus, dietary differences were present, though we do not know to what extent this influenced the results (see Additional file 1). Therefore, though we observed significantly higher fasting and postprandial glucose levels for twins, we cannot ascertain whether this related specifically to the fetal environment or maternal and post-natal nutritional patterns.

In relation to the divergent IGT and IFG findings observed, it is important to note that they may have a different etiology. IFG has been related to raised hepatic glucose output and dysfunction in insulin secretion, whereas IGT is associated with peripheral insulin resistance [20]. Thus, it is possible that twins are at an increased risk of IFG, but not IGT.

Finally, it has been proposed that age is important in unmasking the effect of adverse exposures during fetal life [12]. As our cohort was young, we cannot exclude that over time regulatory deficits in twins - possibly expressed early by higher fasting and two hour glucose levels - will lead to an increased risk of IGT, and subsequently DM.

### Perspectives

The burden of DM is rising rapidly in Africa, though limited data are available about the underlying etiology [45]. If fetal programming is an important component in DM development, the causal pathways may indeed be different in low income African settings [4]. Twin studies represent a powerful tool in diabetes research, as they can control for the contribution of genetic factors [14, 40]. It is therefore relevant to conduct more metabolic twin studies in Africa. Larger studies with older participants are encouraged, particularly those comparing birth weight discordant MZ twin pairs, as a means of evaluating the effect of the intrauterine environment alone, without genetic and post-natal environmental confounding [40].

## Conclusions

Our OGTT-study conducted among twins and singletons in Guinea-Bissau is the first of its kind in Africa, and it provides unique insights into a high mortality population, which has not been investigated before. We found higher blood glucose levels among twins both in the fasting and postprandial states, which could relate to the twin fetal environment. No significant difference was observed in IGT prevalence between the two groups, and the overall burden of dysglycemia was low.

## Abbreviations

BF%, body fat percentage; BMI, body mass index; DM, diabetes mellitus; DZ, dizygotic; HC, hip circumference; HDSS, health and demographic surveillance system; HIV, human immunodeficiency virus; HOMA, homeostasis model assessment; IFG, impaired fasting glucose; IGT, impaired glucose tolerance; INDEPTH, International Network for the Demographic Evaluation of Populations and Their Health in Developing Countries; LBW, low birth weight; MZ, monozygotic; OGTT, oral glucose tolerance test; WC, waist circumference; WHO, World Health Organisation; WHR, waist-to-hip ratio

## Additional file

Additional file 1: Nutritional intake among twins and singletons. (PDF 57 kb)
