# Supplementary material for: Prevalence of impaired glucose tolerance and other types of dysglycaemia among young twins and singletons in Guinea-Bissau
Source: BMC Endocr Disord. 2016 Aug 4;16:46. doi: 10.1186/s12902-016-0126-6 (PMC4973540; doi:10.1186/s12902-016-0126-6)
Supplement: Additional file 1: — Nutritional intake among twins and singletons. (PDF 57 kb) [file 12902_2016_126_MOESM1_ESM.pdf]

BMC Endocrine Disorders.

Title: Prevalence of impaired glucose tolerance and other types of dysglycaemia among young twins and singletons in Guinea-Bissau.

Authors: D. E. Hennild, M. Bjerregaard-Andersen, L. C. Joaquím, K. Christensen M. Sodemann, H. Beck-Nielsen and D. M. Jensen.

Additional file 1: Nutritional intake among twins and singletons.

	**Twins** N = 209	**Singletons** N = 182	**P**
Eats varied food	206/209 (99%)	179/180 (99%)	0.41
Eats breakfast	198/205 (97%)	178/181 (98%)	0.27
Eats fish every day	158/209 (76%)	172/180 (96%)	<0.001
Eats meat every day	3/208 (1.4%)	7/180 (3.9%)	0.13
Eats meat several times a week	87/208 (42%)	95/180 (53%)	0.03
Eats vegetables every day	13/209 (6.2%)	13/180 (7.2%)	0.70
Eats vegetables several times a week	92/209 (44%)	65/180 (36%)	0.11
Eats sweets every day	38/209 (18%)	22/180(12%)	0.10
Eats oily food every day	115/208 (55%)	131/180 (73%)	<0.001
Eats oily food a couple of times a week	92/208 (44%)	47/180 (26%)	<0.001
Eats deep fried food every day	27/208 (13%)	39/180 (22%)	0.02
Eats deep fried food several times a week	138/208 (66%)	93/180 (52%)	0.003
Eats rice every day	207/209 (99%)	181/181 (100%)	0.20
Eats potatoes every day	5/208 (2.4%)	16/179 (8.9%)	0.005
Eats potatoes several times a week	38/208 (18%)	88/179 (49%)	<0.001
Primarily drinks water	204/209 (98%)	177/180 (98%)	0.61
Primarily drinks milk, juice/soda, other	5/209 (2.4%)	3/180 (1.7%)	0.61
